# Supplementary material for: Deposition of Tetracoordinate Co(II) Complex with Chalcone Ligands on Graphene
Source: Molecules. 2020 Oct 29;25(21):5021. doi: 10.3390/molecules25215021 (PMC7662825; doi:10.3390/molecules25215021)
Supplement: Supplementary file 1 [file molecules-25-05021-s001.pdf]

molecules

MDPI

Supplementary Materials

Deposition of tetracoordinate Co(II) complex with chalcone ligands on graphene

Jakub Hrubý [1], Šárka Vavrečková [1,2], Lukáš Masaryk [3], Antonín Sojka [1], Jorge Navarro-Giraldo [1], Miroslav Bartoš [1], Radovan Herchel [3], Ján Moncol [4], Ivan Nemec [1,3] and Petr Neugebauer [1,*]

1 Central European Institute of Technology, CEITEC BUT, Purkyňova 656/123, 61200 Brno, Czech Republic;
2 Institute of Physical Engineering, Faculty of Mechanical Engineering, Brno University of Technology, Technická 2, 61669 Brno, Czech Republic
3 Department of Inorganic Chemistry, Faculty of Science, Palacký University, 17. listopadu 12, 77147 Olomouc, Czech Republic
4 Department of Inorganic Chemistry, Faculty of Chemical and Food Technology, Slovak University of Technology in Bratislava, 81237 Bratislava, Slovakia

* Correspondence: petr.neugebauer@ceitec.vutbr.cz;

Received: date; Accepted: date; Published: date

Table S1. Comparison of Raman shift peaks (in cm^{-1}). Peak intensity is denoted as follows: strong - s, medium - m, weak - w.

Powder Co(II)	CVD graphene	Co(II) on graphene drop-cast	Co(II) on graphene sublimation at 75 °C	Co(II) on graphene sublimation at 265 °C
	301m	304w	307w	
409w				
	430w	435w		
520s	520s	523s	523s	521s
				594w
637w	618w	628w		637w
	669w	674w		
774m				777w
811w	821w			
877w				
895w				897w
964m	946-976m	946-976m	964m	946-976m
984m				989m
1029w			1034w	1036w
1116w		1112w		
				1168m
1186m		1190w	1193m	1190w
1220w			1228w	1224m
1264w			1262w	1261w
1308m			1307m	1306w
	1347m	1346m	1343m	1341m
1366m			1372w	1370w
1425w			1432w	1424w
			1493w	1489w
1529w				1519w
1577m				
	1595m	1591m	1596m	1589s
1603s			1603s	1603s
	1626w			
1656w			1656w	1654w
	2462w	2463w		2463w
	2689m	2682m	2678m	2679m
	2939w	2950w	2945w	2950w
				3022w
				3070w

Figure S1 shows the AFM images with profiles from white lines. The drop-cast sample consists of nanodroplets up to 50 nm high. On the contrary, sublimated samples resulted in the formation of microcrystals hundreds of nanometers high.

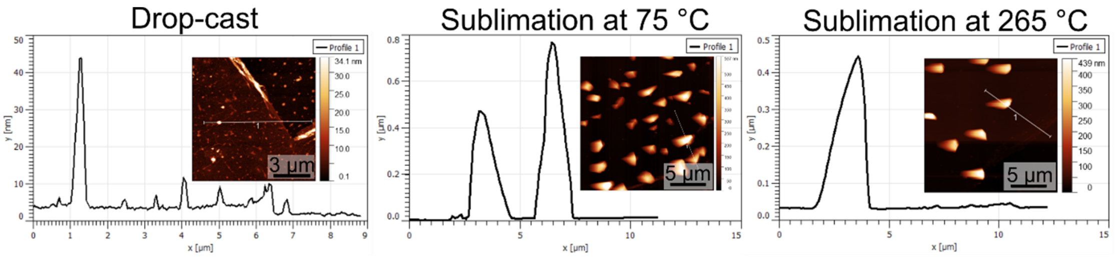

Figure S1. AFM images from drop-cast and sublimated samples at 75 °C and 265 °C.

Figure S2 shows a weak signal from Co 2p found on the surface after drop-cast. The right side of the image shows the fit in the CasaXPS program with a weak Co 2p peak detected.

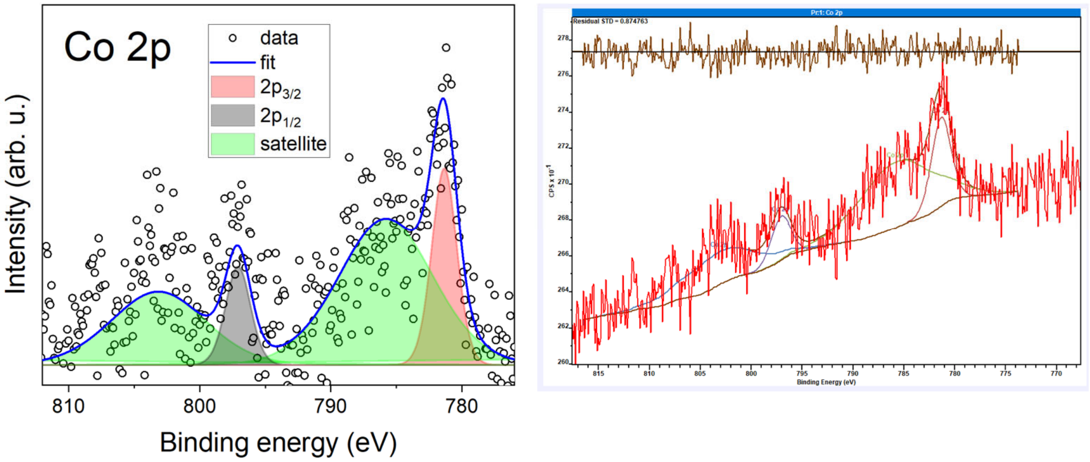

Figure S2. Weak Co 2p peak from the drop-cast sample.

Figure S3 illustrates the semi-empirical quantitative analysis of elemental composition for the studied complex of powder taken from crucible after sublimation at 75 °C and 265 °C. The red line denotes the calculated ideal percental amount of each element from the compound $CoC_{38}H_{32}Cl_2N_4O_4$. Histograms illustrate that cobalt and chlorine content increases as the sublimation temperature rises; this means that cobalt stays in the crucible and supports the observation of no cobalt detected on the surface of microcrystallites after thermal sublimation.

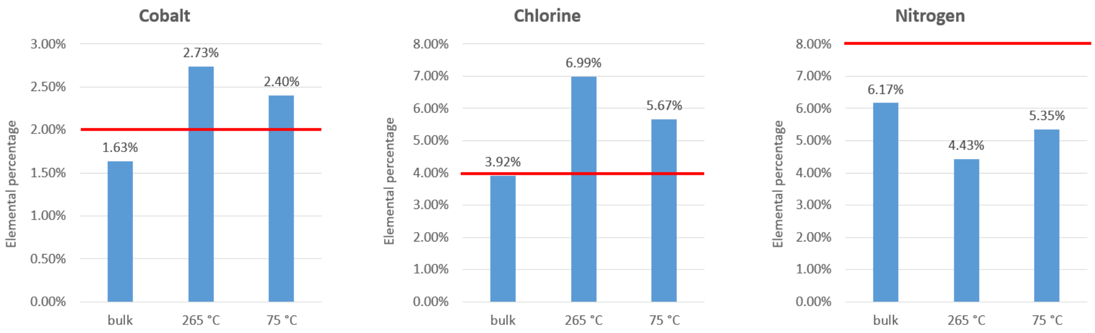

Figure S3. Elemental percentage of atoms in powder from crucible after thermal sublimation at 75 °C and 265 °C.
